# Clade-specific long-read sequencing increases the accuracy and specificity of the *gyrB* phylogenetic marker gene

**DOI:** 10.1128/msystems.01480-24

**Published:** 2024-12-16

**Authors:** Robert G. Nichols, Emily R. Davenport

**Affiliations:** 1 Department of Biology, The Pennsylvania State University, University Park, Pennsylvania, USA; 2 One Health Microbiome Center, Huck Life Sciences Institute, University Park, Pennsylvania, USA; 3 Huck Institutes of the Life Sciences, The Pennsylvania State University, University Park, Pennsylvania, USA; University of California San Diego, La Jolla, California, USA

**Keywords:** GyrB, long-read sequencing, codiversification, cospeciation

## Abstract

Phylogenetic marker gene sequencing is often used as a quick and cost-effective way of evaluating microbial composition within a community. While 16S rRNA gene sequencing (16S) is commonly used for bacteria and archaea, other marker genes are preferable in certain situations, such as when 16S sequences cannot distinguish between taxa within a group. Another situation is when researchers want to study cospeciation of host taxa that diverged much more recently than the slowly evolving 16S rRNA gene. For example, the bacterial gyrase subunit B (*gyrB*) gene has been used to investigate cospeciation between the microbiome and various hominid species. However, to date, only primers that generate short-read Illumina MiSeq-length amplicons exist to investigate *gyrB* of the Bacteroidaceae, Bifidobacteriaceae, and Lachnospiraceae families. Here, we update this methodology by creating *gyrB* primers for the Bacteroidaceae, Bifidobacteriaceae, and Lachnospiraceae families for long-read PacBio sequencing and characterize them against established short-read *gyrB* primer sets. We demonstrate both bioinformatically and analytically that these longer amplicons offer more sequence space for greater taxonomic resolution, lower off-target amplification rates, and lower error rates with PacBio CCS sequencing versus established short-read sequencing. The availability of these long-read *gyrB* primers will prove to be integral to the continued analysis of cospeciation between bacterial members of the gut microbiome and recently diverging host species.

**IMPORTANCE:**

Previous studies have shown that the marker gene gyrase subunit B (*gyrB*) can be used to study codiversification between the gut microbiome and hominids. However, only primers for short-read sequencing have been developed which have limited resolution for subspecies assignment. In the present study, we create new *gyrB* primer sets for long-read sequencing approaches and compare them to the existing short-read *gyrB* primers. We show that using longer reads leads to better taxonomic resolution, lower off-target amplification, and lower error rates, which are vital for accurate estimates of codiversification.

## INTRODUCTION

Metabarcoding methods have made it possible to study a variety of microbiomes, from environmental to human body sites. These techniques involve sequencing phylogenetic marker genes to characterize the bacteria and archaea present in a microbiome, independent of culturing. The most widely used marker gene for bacteria and archaea is the 16S ribosomal RNA (rRNA) gene (1, 2). The 16S rRNA gene is an attractive marker sequence because it is present in all prokaryotes and archaea (3) and includes conserved regions that can be used for primer generation and multiple hypervariable regions that contain species-specific sequences (4). Sequencing of the 16S rRNA gene can provide very cheap, rapid, and accurate taxonomic identification of the members of a microbiome.

Currently, the most popular way to sequence the 16S rRNA gene amplicons is with short-read sequencing technology, like the Illumina MiSeq. Illumina MiSeq sequencing is well validated and very high throughput and can provide reliable taxonomic information down to the genus level. Unfortunately, 16S Illumina MiSeq sequencing cannot provide species or strain-based resolution (5). To achieve this level of specificity, one would need to sequence the entire 16S rRNA gene (5). The 16S gene is only ~1,550 base pairs (bp), which makes it an ideal target for long-read sequencing with PacBio technology (1). Along with being able to sequence the entire 16S gene, PacBio technology boasts a potential error rate of 1 bp per million bases after consensus is reached (6). This error rate is much lower than the reported error rate of 1 out of 1,000 bases from Illumina (7). Having the lowest error rate possible is essential for investigating bacterial strain level differences.

The ability to generate long reads and low error rates is also important for other, non-16S rRNA, marker genes that can be used to investigate other characteristics of a microbiome like evolution and coevolution. One such target is the bacterial DNA gyrase subunit B gene (*gyrB*), which has been validated and used in studies that require strain and subspecies resolution (8). This is an attractive marker gene because unlike 16S, there is only one copy of *gyrB* in each bacterial species (9). Additionally, there are low rates of *gyrB* gene transfer between bacterial species in a microbiome (9), and there is a greater degree of divergence in *gyrB* within and between species when compared other marker genes, like 16S (10). This makes *gyrB* an ideal marker gene for studies investigating microbiome evolution and coevolution on relatively short time scales (~millions of years).

For example, a recent study by Moeller et al. (11) uses bacterial *gyrB* to investigate cospeciation between hominids and three bacterial families within their gut microbiomes: Bacteroidaceae, Bifidobacteriaceae, and Lachnospiraceae. Specifically, the PhyloTAGs software (12) was used to create family-specific primers to amplify the bacterial *gyrB* gene from human, chimp, bonobo, and gorilla fecal samples (11). These amplicons were then sequenced on the Illumina MiSeq and used to create maximum likelihood trees to investigate which bacterial families coevolved with hominids. This short-read data provided a proof of concept that there are potential cospeciation relationships between the bacteria of the gut microbiome and hominid hosts. Current short-read amplicon sequencing has technological limitations involving sequencing length and error rates which potentially limit the specificity and accuracy of these relationships. More recent codiversification studies have moved toward using metagenomic data to study those relationships (13–15). With metagenomic data, very deep, expensive sequencing is needed to gain accurate, strain-level resolution across the entire microbiome. This is especially prohibitive for rare or lowly abundant taxa, where high sequencing depths would be needed to accurately estimate abundances of those subspecies. Targeting specific families with amplicon-based sequencing would allow for complete and accurate characterization of even these lowly prevalent taxa. To further refine these relationships, updated methodology is needed that will account for these current limitations.

Sequencing the *gyrB* marker gene with PacBio circular consensus sequencing (CCS) offers two major advantages over short-read sequencing. First, as mentioned above, the ability for PacBio to sequence the full-length gene proves a greater taxonomic resolution when compared to short-read sequencing, which can only sequence a part of the gene (16). The relatively small gene size of *gyrB* (average 2,000–2,500 bp) makes *gyrB* an ideal target for long-read PacBio sequencing. Second, PacBio CCS typically results in a much lower error rate than what is obtained with short-read sequencing (1 out of a million bp vs 1 out of 1,000 bp) (6, 7). These lower error rates are necessary for cospeciation studies where single-nucleotide variations are used to track bacterial evolution. Studies have already shown that PacBio sequencing of similar-sized genes like the 16S gene (1,550 bp) has error rates close to 0% and produces reads at a single-nucleotide resolution (17). Unfortunately, unlike the 16S gene, there are currently no primers available for long-read *gyrB* sequencing.

In the present study, we generated and tested three new *gyrB* primer sets designed for PacBio sequencing for the Bacteroidaceae, Bifidobacteriaceae, and Lachnospiraceae bacterial families. Like the original Moeller study, we used the PhyloTAGs framework to design long-read primers targeted for these three families. We identified primers for each family with the lowest predicted off-target effects. Each primer set was used to generate PacBio sequencing data for several human fecal samples, murine cecal samples, and a mock community. We then compared these results to those obtained with the original short-read primer sets on the same samples. Overall, we were able to see better resolution, less off-target amplifications, and greater accuracy with the long-read *gyrB* primers when compared to the original short-read *gyrB* primers.

## MATERIALS AND METHODS

### Creation of long-read *gyrB* primers with PhyloTAGs

To create the new long-read *gyrB* primers for Bacteroidaceae, Bifidobacteriaceae, and Lachnospiraceae families (which will be called the “Nichols” primers going forward), the existing tool PhyloTAGs was used (12). PhyloTAGs creates primers to amplify a specific gene at a desired level of phylogenetic specificity. A reference database, a 16S rRNA gene (16S) identity map, and a reference organism are needed to use PhyloTAGs. For this project, the reference database includes all the bacterial *gyrB* genes (“gyr_refseq.fa”; *n* = 31,842 genes in 31,842 bacteria; available on Zenodo, DOI 10.5281/zenodo.10451935) extracted from the National Center for Biotechnology Information (NCBI) RefSeq non-redundant (nr) database downloaded on 10 April 2023 (“Bacteria.refseq.tar.gz.part1”; available on Zenodo, DOI 10.5281/zenodo.10452184 and “Bacteria.refseq.tar.gz.part2”; DOI 10.5281/zenodo.10452279). Along with the bacterial *gyrB* genes, 16S sequences were extracted from the same species used to create the 16S database (“16S_refseq.fa”; *n* = 31,842 genes in 31,842 bacteria; available on Zenodo, DOI 10.5281/zenodo.10451935). A non-redundant version of the corresponding 16S sequences was used to create a 16S rRNA gene identity map with the USEARCH program (18). This identity map gives a percent identity value for every bacterium used in the database, compared to all other used bacteria. A reference organism is chosen from the bacteria present in the 16S/*gyrB* database based on which bacterial family the primers are being created. For this project, NZ_CP036539 was used as a reference for Bacteroidaceae, NC_008618.1 was used as a reference for Bifidobacteriaceae, and NZ_LR699005 was used as a reference for Lachnospiraceae.

PhyloTAGs requires an “idlow” and an “idhigh” value for primer specificity, to indicate sequence similarity cutoffs for primer generation. For this project, an “idhigh” value of 100 was used for each family. Since sequence similarities of a given taxonomic level differs across the phylogenetic tree (19, 20), we determined “idlow” values for each family separately. The “idlow” values were determined by using PhyloTAGs at various values and testing resulting primers with an *in silico* PCR approach with the USEARCH program (18). A human microbiome data set from the NCBI (https://ftp.ncbi.nlm.nih.gov/genomes/HUMAN_MICROBIOM/; downloaded 8 August 2022) was used for a reference database to test the primers. This database includes ~950 bacterial species specific to the human microbiome, making it an appropriate test database for the primers of interest. Primers with the least amount of off-target amplification were chosen. Ultimately, “idlow” values of 91 for Bacteroidaceae, 95 for Bifidobacteriaceae, and 92 for Lachnospiraceae were selected.

To generate the final candidate primer list, PhyloTAGs was run using the “idhigh” and “idlow” values specified above, a codons value of 7, a consensus value of 90, and the greedy option. The codons value determines the length of the created primers; in this case, we are using seven codons or 21 bp in length. The consensus option is the percentage required for a single nucleotide to be used in the primer sequence. Consensus values that fall outside this range get the corresponding IUPAC nucleotide code; for example, Y is used for a nucleotide that could be C or T. The greedy option is used to explore the entire *gyrB* gene when looking for primers. The primer sequences, degeneracy values, and position windows are reported from PhyloTAGs (Tables S1 to S3). Ultimately, we chose two primer sets per family based on low degeneracy values (degeneracy values 96 or lower) to test with *in silico* PCR. The best-performing primer set (least number of off-target amplifications) was then validated in the lab (Table 1).

**TABLE 1:** *gyrB* primers and amplicon band sizes^*a*^

	Forward primer	Reverse primer	Amplicon size (bp)
Moeller Bacteroidaceae	*TCGTCGGCAGCGTCAGATGTGTATAAGAGACAG* CGGAGGTAARTTCGAYAAAGG	*GTCTCGTGGGCTCGGAGATGTGTATAAGAGACAG* GCRTATTTYTTCARHGTACGG	608
Moeller Bifidobacteriaceae	*TCGTCGGCAGCGTCAGATGTGTATAAGAGACAG* GACRACGGNCGNGGCATYCC	*GTCTCGTGGGCTCGGAGATGTGTATAAGAGACAG* AGNCCCTTGTTNAGGAAVGCC	395
Moeller Lachnospiraceae	*TCGTCGGCAGCGTCAGATGTGTATAAGAGACAG* GGHGGAGGATAYAAGGTATCC	*GTCTCGTGGGCTCGGAGATGTGTATAAGAGACAG* TRTANGAATCRTTRTGCTGC	502
Nichols Bacteroidaceae (NPB-Bacter)	*/5AmMC6/GCAGTCGAACATGTAGCTGACTCAGGTCAC* TGTAYATYGGTGACATYAGYR	*/5AmMC6/TGGATCACTTGTGCAAGCATCACATCGTAG* CCCATYARCATRGARAAGATR	1800
Nichols Bifidobacteriaceae (NPB-Bifido)	*/5AmMC6/GCAGTCGAACATGTAGCTGACTCAGGTCAC* ATCGARGTSACGATTCTGCCG	*/5AmMC6/TGGATCACTTGTGCAAGCATCACATCGTAG* GGATCCATGGTGGTYTCCCAC	1678
Nichols Lachnospiraceae (NPB-Lachno)	*/5AmMC6/GCAGTCGAACATGTAGCTGACTCAGGTCAC* SAGRGGWCTBCATCATYTRGT	*/5AmMC6/TGGATCACTTGTGCAAGCATCACATCGTAG* TCMGGATCCATDGTBGTCTCC	1673

^*a*^ “Moeller” short-read amplicon primer sets were originally described in Moeller et al. (11), while the “Nichols” long-read amplicon primer sets are described here. In the forward and reverse primer columns, italicized text shows either the Illumina overhang (Moeller primers) or the PacBio overhang (Nichols primers) needed for sequencing, while nonitalicized text shows the degenerate primers as determined by PhyloTAGs. /5AmMC6/is a modification needed for PacBio library prep.

### DNA isolation

Five human fecal samples obtained from BioreclamationIVT (now BioIVT), two C57BL/6 wild-type murine cecal samples (generated previously in [21]), and one mock community (ZymoBIOMICS Gut Microbiome Standard, Zymo Research, Irvine, CA, USA) were used to test the efficacy of the primer sets across multiple sample types. DNA was isolated from the eight test samples using the Omega Bio-tek E.Z.N.A Stool DNA Kit (Omega Bio-tek, Norcross, GA, USA) using the manufacturer-recommended protocol. DNA quantity and quality were measured via a NanoDrop One Spectrophotometer (Thermo Scientific, Waltham, MA, USA).

### *gryB* amplification and sequencing

**Fig 1:**
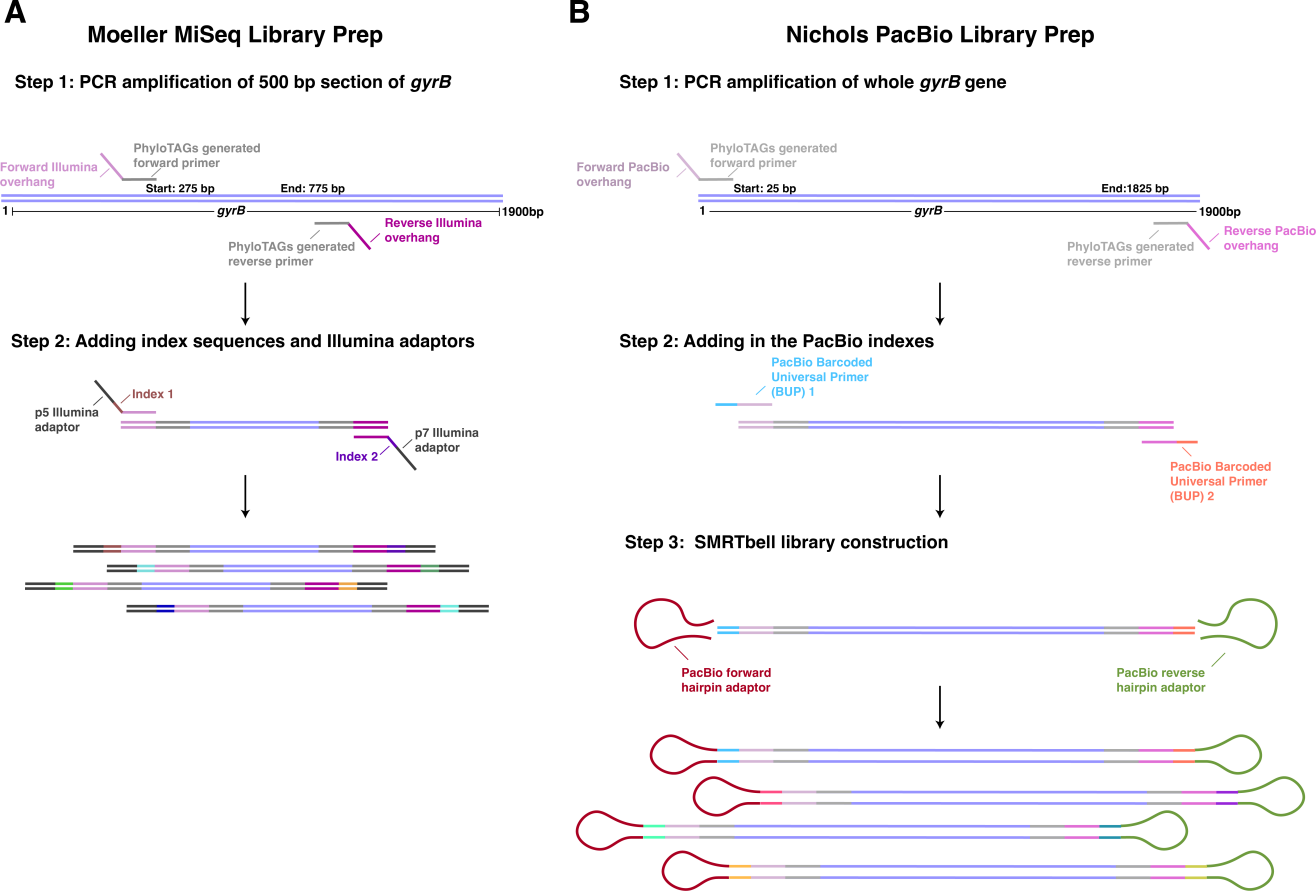
Library preparation schemes for MiSeq and PacBio. (**A**) Library preparation scheme for MiSeq sequencing of *gyrB* amplicons using the Moeller short-read primers. Briefly, family-specific primers (gray) with Illumina overhang sequences (pink/purple) are used to amplify a ~500 bp amplicon from the *gyrB* gene. In a second step, Illumina p5/p7 adapters (dark gray) and index sequences (multi-colored) are added to each sample. (**B**) Library preparation scheme for the PacBio sequencing of the *gyrB* amplicons using the Nichols long-read primers. Briefly, family-specific primers (gray) with PacBio adapter sequences (light/dark pink) are used to amplify an ~1,800-bp amplicon from the *gyrB* gene. In step 2, the PacBio barcoded universal primers are added to either end (pink/blue and dark pink/salmon) of each amplicon. Finally, in step 3, the PacBio hairpin adapters (maroon/green) are added to circularize the fragments for CCS. Ultimately, each library preparation scheme results in amplicons indexed by sample that can be multiplexed for sequencing.

Detailed protocols for 16S and *gyrB* PacBio library preparation are available on protocols.io (dx.doi.org/10.17504/protocols.io.36wgq31nylk5/v1). Three clade-specific long-read PacBio primer sets (“Nichols”, generated here) and three short-read primer sets (“Moeller”, previously published [11]) were used to amplify *gyrB* of Bacteroidaceae, Bifidobacteriaceae, and Lachnospiraceae as these were the three families previously investigated by Moeller et al. (Table 1). DNA from the five human samples (H1–H5), two murine samples (M1 and M2) and mock sample (GM) described above underwent library preparation for *gyrB* sequencing. PCR mixtures contained Platinum SuperFi DNA Polymerase Master Mix (Invitrogen), 0.4 M of both forward and reverse primers, and 10 ng of template DNA for each sample. PCR conditions for the PacBio *gyrB* amplifications were 30 s at 95°C, 30 cycles of 30 s at 95°C, 30 s at 57°C and 1 min at 72°C, and then 5 min at 72°C. PCR conditions for the original Moeller MiSeq *gyrB* amplifications were 2 min at 98°C, 25 cycles of 10 s 98°C, 20 s 56.6°C, 15 s 72°C, and then one cycle of 5 min at 72°C, as previously described (22). PCRs were cleaned up using SPRI magnetic beads (Clean NGS) as described in the manufacturer-provided instructions. Samples were then run on a 1× agarose gel to visualize correct amplification size, between 1,600 and 1,800 bp for PacBio amplicons and between 300 and 700 bp for MiSeq amplicons (Table 1). DNA was transferred to the Pennsylvania State Genomics Core facility where amplicon size selection was performed with a BluePippin (Sage Science, Beverly, MA, USA), indexes were added on to each sample, and then samples were mixed in equimolar amounts using SequalPrep Normalization Plates (ThermoFisher Scientific, Waltham MA). The libraries created with the original Moeller primers were sequenced on the Illumina MiSeq with 250 × 250 paired-end sequencing using Reagent Kit v2, resulting in ~310,000 reads per sample. The amplicons created with the new Nichols PacBio primers were sequenced on the PacBio Sequel lle using the SMRTbell Express Template Prep Kit 2.0 and the Sequel II Binding Kit, resulting in ~100,000 reads per sample. The library prep schemes for both PacBio and Illumina MiSeq amplicons can be seen in Fig. 1.

### 16S amplification and sequencing

16S sequencing of the five human samples (H1–H5), two murine samples (M1 and M2), and mock sample (GM) was also performed on both the Illumina MiSeq and PacBio Sequel IIe. PCR mixtures contained Platinum SuperFi DNA Polymerase Master Mix (Invitrogen), 0.4 M of both forward and reverse primers, and 10 ng of template DNA for each sample. Traditional long-read 16S primers (27F: AGRGTTYGATYMTGGCTCA and 1492R RGYTACCTTGTTACGACTT) were used for PacBio sequencing (23). PCR conditions for the PacBio 16S amplifications were 30 s at 95°C, 30 cycles of 30 s at 95°C, 30 s at 57°C, 1 min at 72°C, and then 5 min at 72°C. For MiSeq sequencing, V4 primers were used for 16S sequencing (515F: GTGYCAGCMGCCGCGGTAA and 806R: GGACTACNVGGGTWTCTAAT) (24). PCR conditions for the MiSeq 16S samples were 2 min at 98°C, 25 cycles of 10 s 98°C, 20 s 56.6°C, 15 s 72°C, and then 1 cycle of 5 min at 72°C (22). PCRs were cleaned up using SPRI magnetic beads (Clean NGS) as described in the manufacturer-provided instructions. Samples were then run on a 1× agarose gel to visualize correct amplification size, ~1,400 bp for PacBio 16S amplicons and ~350 bp for MiSeq 16S amplicons. DNA was transferred to the Pennsylvania State Genomics Core facility where amplicon size selection was performed with a BluePippin (Sage Science, Beverly, MA, USA), indexes were added on to each sample, and then samples were mixed in equimolar amounts using SequalPrep Normalization Plates (ThermoFisher Scientific, Waltham MA, USA). The libraries created with the V4 primers were sequenced on the Illumina MiSeq with 250 × 250 paired-end sequencing using Reagent Kit v2 with the *gyrB* MiSeq samples (described above), resulting in ~310,000 average reads per sample. The long-read 16S amplicons were sequenced on the PacBio Sequel lle with the *gyrB* PacBio samples (described above) using the SMRTbell Express Template Prep Kit 2.0 and the Sequel II Binding Kit, resulting in ~100,000 average reads per sample.

### Analysis of *gyrB* sequencing reads with DADA2

Both the Illumina and PacBio *gyrB* reads were analyzed with DADA2 (v 1.26) (25). Briefly, for the PacBio data, we used minQ = 3, minLen = 1,000, maxLen = 2,000, maxN = 0, rm.phix = FALSE, and maxEE = 3 for the filter and trim step. Samples were then dereplicated and denoised with an error rate created from the samples. Chimeras were then removed with the consensus method and samples were classified with the custom *gyrB* reference database that used in primer creation (“NCBI_nr_gyrB”). Unclassified reads were assigned with BLASTX and the RefSeq NR database to obtain taxonomy. The top hit for each read with a percent identity above 75% was used for taxonomy assignment. BLASTX results with a percent identity of less than 75%, or a non-*gyrB* gene assignment resulted in an “Unclassified” taxonomy assignment. For the MiSeq data, reads were filtered with truncLen = c(250,230), maxN = 0, maxEE = c (2, 2), truncQ = 2, rm.phix = TRUE, and trimLeft being variable, depending on the size of the primers; see GyrB_dada2_analysis.Rmd in the GitHub repository at https://github.com/davenport-lab/Long-read_gyrB_method. Samples were used to create an error rate and then denoised and merged as described above. Chimeras were removed from the merged reads. Like the PacBio reads, the MiSeq reads were classified with the custom *gyrB* reference database (“NCBI_nr_gyrB”). Unclassified reads were assigned with BLASTX, with results at a percent identity of less than 75% or a non-GyrB protein assignment resulted in an “Unclassified” taxonomy assignment. In addition, MiSeq reads were also classified with the corresponding PacBio reads as a database to check for overlap.

### Creation of pseudo-MiSeq sequences and comparison to PacBio ASVs

PacBio *gyrB* reads were cut using the Moeller short-read primers and the *in silico* PCR option with the USEARCH program (18). The resulting cut reads were treated as “pseudo-MiSeq” amplicons from the same samples, to mimic the specificity that a shorter amplicon would provide. These cut reads were analyzed the same way as the original PacBio reads described above. In addition to classifying these reads with the custom *gyrB* reference database (“NCBI_nr_gyrB”), the pseudo-MiSeq reads were also classified with the original parent PacBio ASVs. This second classification was done to match the pseudo-Miseq read with its parent PacBio ASV read. In some cases, the pseudo-Miseq read matched to multiple parent PacBio ASVs, and this resulted in a “NA” classification. These reads were individually searched and matched based on similar read count data between the parent PacBio ASV and the pseudo-Miseq ASV. Additionally, some low abundance (<0.1% total abundance) pseudo-MiSeq reads did not have a parent PacBio ASV match; these were denoted as “unassigned.”

The pseudo-MiSeq ASV sequences and their matching Pacbio ASV sequences were then individually aligned using the ClustalW algorithm within the MEGA 11 program (26). After alignment, maximum likelihood trees were created with MEGA 11 using the general time reversible model, including invariant sites and used the nearest neighbor interchange heuristic method (26). The resulting trees were then imported to dendroscope to create tanglegrams to visualize matches of pseudo-MiSeq sequences with their corresponding parent PacBio sequence (27). The environment for tree exploration toolkit was used to calculate the normalized Robinson–Foulds (RF) symmetric difference between each tree pair (parent PacBio ASVs and pseudo-MiSeq ASVs) (28).

## RESULTS

To generate the ideal long-read sequencing amplicons of *gyrB* for phylogenetic comparisons, we first designed multiple sets of primers per bacterial family considered. We evaluated those primer pairs *in silico* for off-targets effects, and then the best performing pairs were validated experimentally using human and murine cecal samples as well as a mock community of known composition (ZymoBIOMICS Gut Microbiome Standard). These results were then compared to data generated on the MiSeq using the Moeller short-read primers. For each bacterial family examined (Bacteroidaceae, Bifidobacteriaceae, and Lachnospiraceae), we first identified the top-performing long-read primer pair. We then showed that the long-read primers demonstrated better phylogenetic resolution when compared to the corresponding short-read primer pair for that family.

### PhyloTAGs primer locations are chosen based on low degeneracy locations

**Fig 2:**
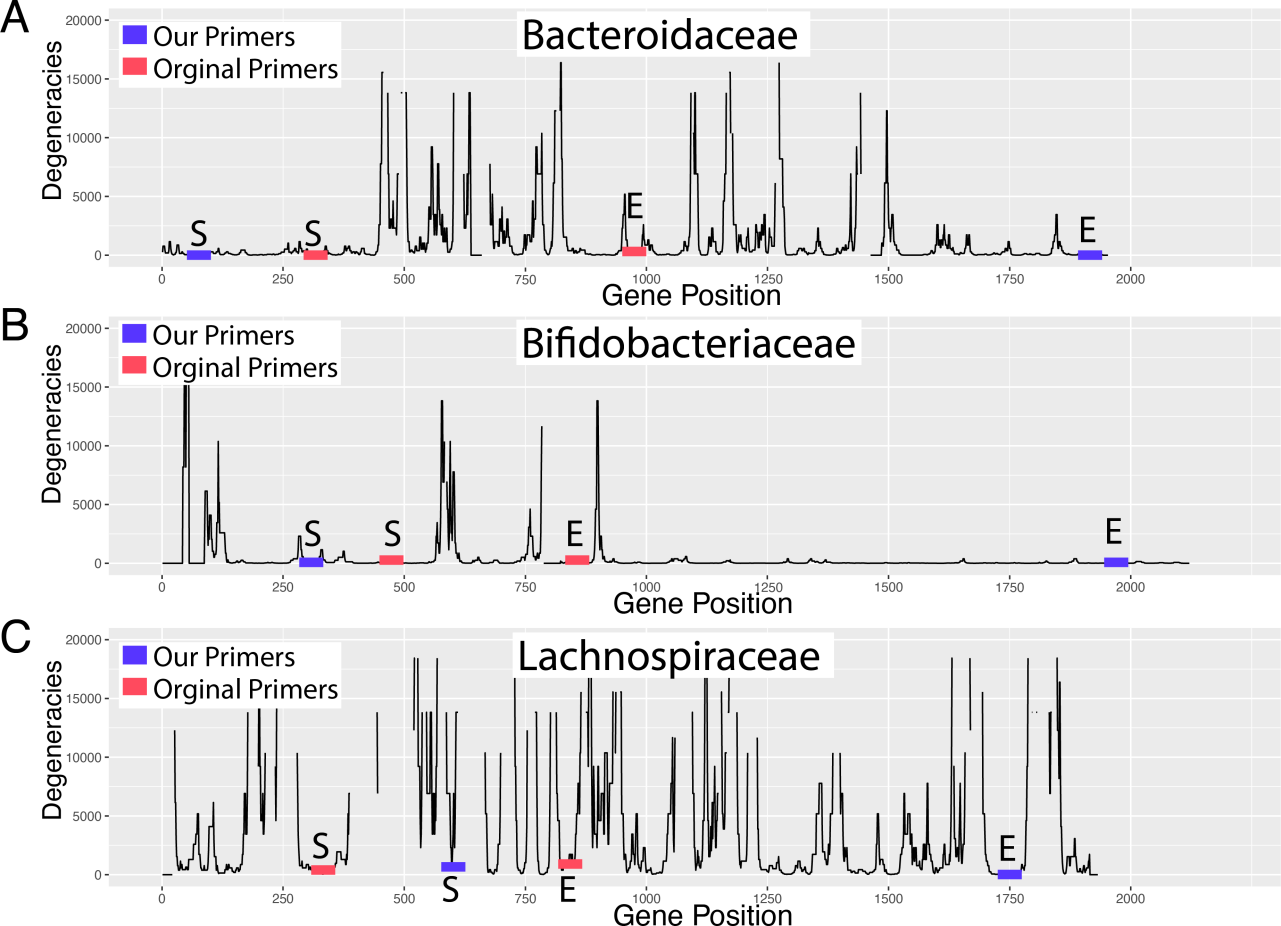
Degeneracy plots showing the genetic coordinates of the Nichols long-read and Moeller short-read *gyrB* primers. We developed long-read, clade-specific primers for the bacterial families (A) Bacteroidaceae, (B) Bifidobacteriaceae, and (C) Lachnospiraceae for use with PacBio sequencing (blue). They target a longer region of the *gyrB* gene (*x*-axis) in each family to capture more of the degeneracies between strains (*y*-axis) than primers designed for short-read sequencing (red). Number of degeneracies per site is indicated on the *y*-axis for each family. S = start, E = end.

We ran PhyloTAGs on each bacterial family to generate a list of candidate long-read primers (see Methods). The results from PhyloTAGs were collapsed based on the degeneracy score to show the locations with the lowest degeneracy values (Fig. 2). Nichols long-read primers were chosen in spots of low degeneracy to give the longest possible read with the fewest potential predicted off-target amplifications (Table 1). For example, the Bifidobacteriaceae degeneracy plot has a low degeneracy primer region that could result in a longer read (Fig. 2B), but after *in silico* PCR, it was determined that the indicated primer locations resulted in the fewest off-target amplifications.

**Fig 3:**
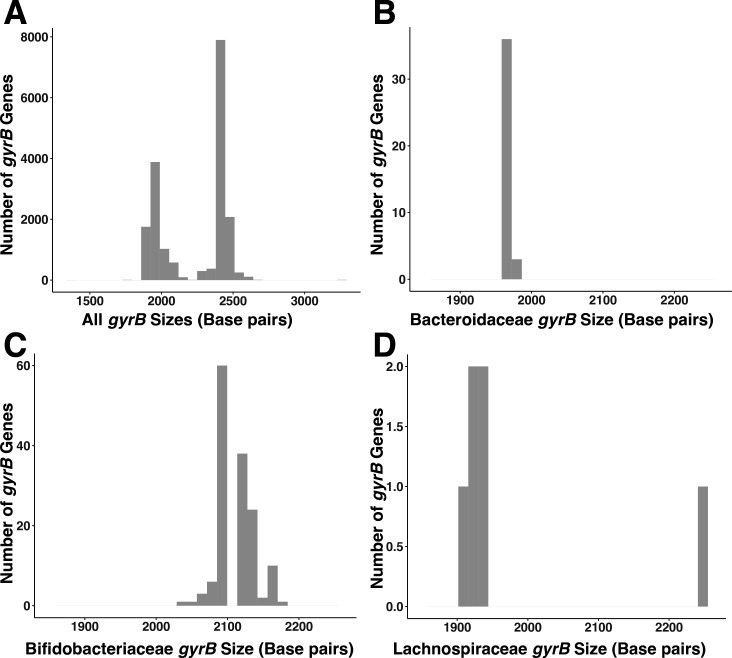
Range of *gyrB* gene sizes within the *gyrB* reference database per family. The length of the *gyrB* gene varies across taxa included in the *gyrB* reference database (A), including the Bacteroidaceae (B), Bifidobacteriaceae (C), and Lachnospiraceae (D) families.

It should be noted that *gyrB* gene length varies both between and within species. Within our complete assembled database which spans the full bacterial phylogeny, lengths range from 1,352 bp to 3,246 bp, with the average size ranging from 2,000 bp to 2,500 bp (Fig. 3). Bacterial *gyrB* also varies in length within each of the three bacterial families utilized for this study. In Bacteroidaceae (*n* = 39 representative species), *gyrB* size ranges from 1,959 bp to 1,974 bp, with the average size being between 1,959 bp and 1,962 bp. Bifidobacteriaceae had the most number of representative species in the *gyrB* database (*n* = 146) and had *gyrB* sizes ranging from 2,040 bp to 2,172 bp and an average length of 2,091 bp. Lachnospiraceae had the least number of representative species in the *gyrB* database (*n* = 6) and had *gyrB* lengths that varied between 1,911 bp and 2,244 bp, with an average size between 1,911 bp and 1,938 bp.

### PacBio *gyrB* ASVs result in higher specificity and accuracy than the pseudo-MiSeq *gyrB* ASVs

Theoretically, the longer sequencing lengths and higher fidelity of PacBio reads (created from the Nichols long-read primers) compared to shorter Illumina reads (created from the Moeller short-read primers) should result in both more sequence space and more accurate variant calls for phylogenetic tree construction. To demonstrate that this is the case, we amplified and sequenced *gyrB* amplicons from a mock community (GM), human fecal samples (H1–H5), and murine samples (M1 and M2) using both the Nichols long-read and Moeller short-read primer pairs (see Methods).

**Fig 4:**
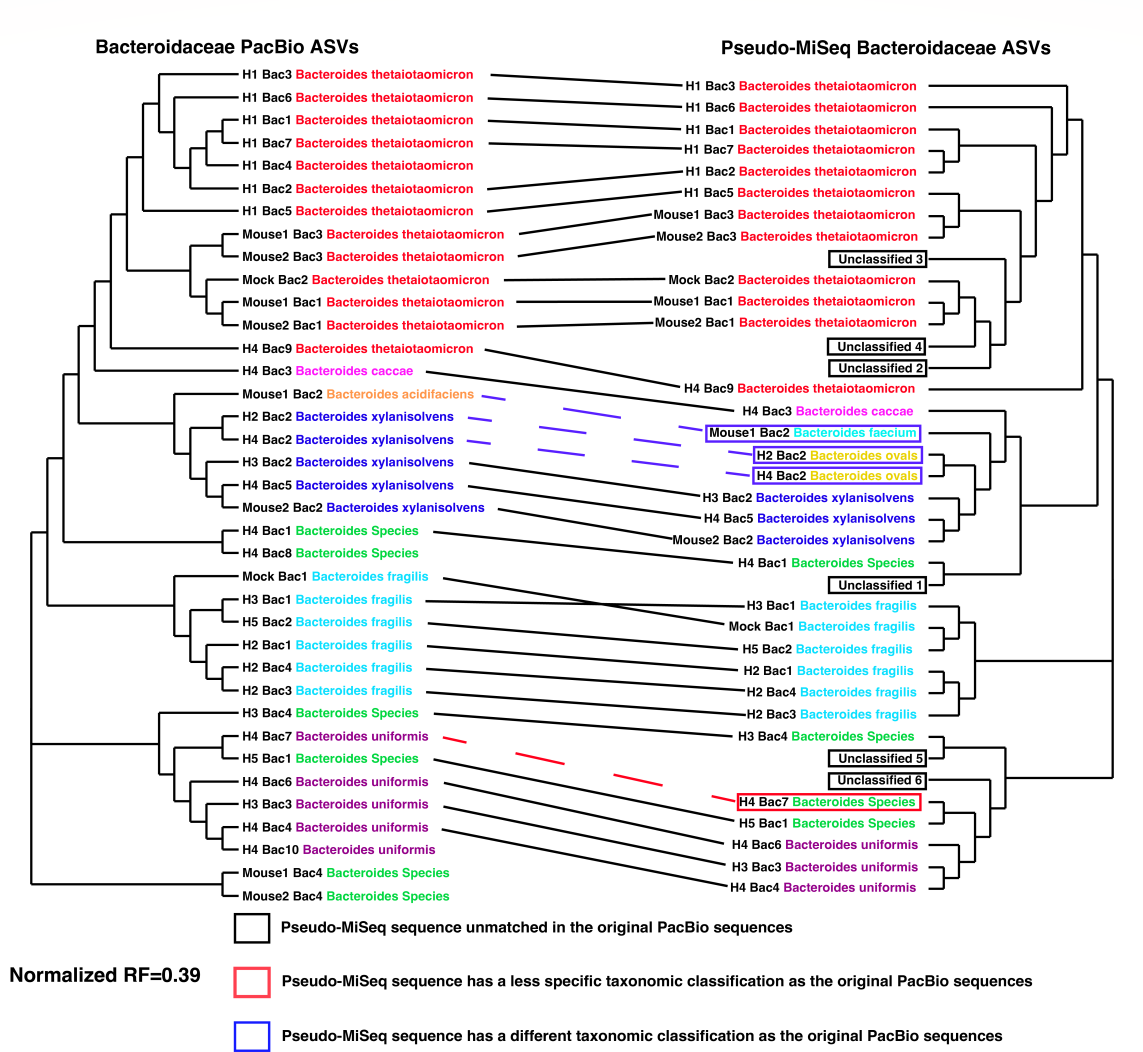
Tanglegram of *gyrB* maximum likelihood trees comparing the Bacteroidaceae PacBio ASVs and the “pseudo-MiSeq” ASVs. Bacteroidaceae phylogeny was generated using the maximum likelihood (ML) method on ASV data collected across all samples. ML trees for long-read PacBio ASVs (left) and short-read pseudo-MiSeq ASVs (right) were compared with a tanglegram. Each tip is annotated with the sample it originated from, the ASV number, and the taxonomic information after classification. ASV classification names are colored by species. The solid black lines connecting the two trees illustrate a one-to-one relationship where the pseudo-MiSeq ASV results in the same classification as the PacBio parent read. The red dashed line illustrates a scenario where the pseudo-MiSeq sequence has a less specific classification than the parent PacBio sequence. The blue dashed line represents conflicting classifications between the pseudo-MiSeq ASV and its parent PacBio ASV. The normalized RF distance for these two Bacteroidaceae trees was 0.39.

First, to establish the improved performance of the high-fidelity long-reads, we compared the amplicons generated via PacBio sequencing to what we call “pseudo-MiSeq” reads. The pseudo-MiSeq reads were generated by trimming each raw PacBio read to its corresponding MiSeq read by using *in silico* PCR and the Moeller short-read primers (11). The resulting tanglegram for the Bacteroidaceae family (Fig. 4) shows good concordance between the long PacBio and pseudo-MiSeq short read, with 28 out of 38 pseudo-MiSeq ASVs classifying to the same bacterial species as their parent PacBio ASV and a normalized RF (nRF) distance of 0.39. Of the 10 pseudo-MiSeq ASVs that do not match to their parent PacBio ASVs, six (or 15.7% of the total ASVs) are unassigned, three (or 7.9% of the total ASVs) classify to a different bacterial species than their parent PacBio ASVs, and one (or 2.6% of the total ASVs) is classified at a less specific taxonomic level than their parent PacBio ASVs (Fig. 4).

There is also somewhat decent concordance between the long PacBio and pseudo-MiSeq short reads for Bifidobacteriaceae, with 10 out of 22 pseudo-MiSeq ASVs classifying to the same bacterial species as their parent PacBio ASV and a nRF of 0.33 (Fig. S1). Of the 12 ASVs that do not match their parent PacBio ASVs, five (or 22.7% of the total ASVs) are unassigned, five (or 22.7% of the total ASVs) classify to a different bacterial species than their parent PacBio ASVs, and two (or 9% of the total ASVs) are classified at a less specific taxonomic level than their parent PacBio ASV.

Lachnospiraceae shows the least concordance between the long and pseudo-MiSeq short reads, with 36 out of 59 pseudo-MiSeq ASVs classifying to the same bacterial species as their parent PacBio ASV and a nRF of 0.40 (Fig. S2). Of the 23 ASVs that do not match their parent PacBio ASVs, nine (or 15.2% of the total ASVs) are unassigned, 12 (or 20.3% of the total ASVs) classify to a different bacterial species than their parent PacBio ASVs, and two (or 3.3% of the total ASVs) are classified at a less specific taxonomic level than their parent PacBio ASV.

Overall, the amplicons generated from Nichols long-read primers resulted in more specific taxonomic assignments when compared to the pseudo-MiSeq sequences. In all three bacterial families tested, we saw unassigned ASVs, incorrect taxonomic assignment of ASVs, and less specific taxonomic assignments for the pseudo-MiSeq ASVs when compared to their parent PacBio amplicons. These discrepancies were most visible in the Lachnospiraceae family which had a nRF = 0.40, followed by Bacteroidaceae with a nRF = 0.39, and then Bifidobacteriaceae with a nRF of 0.33.

### Improved performance of accurate, long-read *gyrB* amplicons vs. short-read *gyrB* amplicons

The pseudo-MiSeq read analysis above demonstrates that the added amplicon length obtained with the PacBio reads provides useful phylogenetic information compared to short reads of equal quality. To further assess the breadth and specificity of the amplicons, as well as the effect of sequencing accuracy, we amplified the mock (GM), human (H1–H5), and murine (M1 and M2) samples described above using the Moeller short-read primers and sequenced using Illumina MiSeq technology (11). We first assessed the extent of off-target amplification using each primer set (Table 2). Table 2 has also been rarified to 28,000 reads per sample in Table S4. The Moeller short-read primers for Bacteroidaceae resulted in 82 ASVs across all eight samples. Of the 82 ASVs, 55 ASVs (98.91% of total reads) classified to Bacteroidaceae (Table 2; Fig. S3; Table S5), for an off-target rate of 1.0%. The Nichols long-read Bacteroidaceae primers resulted in 27 ASVs (100% of total QC reads) across the same samples with all 27 ASVs classifying to Bacteroidaceae and a total off-target rate of 0% (Table 2; Fig. S4; Table S5). Therefore, the proportion of usable, on-target data were much higher with the new long-read primers (100%) vs. the established short-read primers (98.97%) for Bacteroidaceae.

**TABLE 2:** Performance of each primer set across samples^*a*^

Primer set	Total ASVs	On-target ASVs	Off-target ASVs	Total reads	Total QC reads	On-target reads	Off-target reads	Off-target rate
Moeller Bacteroidaceae	82	55	27	913,974	812,985	804,113	8,872	1.09%
Nichols Bacteroidaceae	31	31	0	573,128	346,547	346,547	0	0.00%
Moeller Bifidobacteriaceae	256	28	228	2,087,611	707,401	610,610	96,791	13.68%
Nichols Bifidobacteriaceae	66	26	40	5,58,651	262,096	259,754	2,342	0.89%
Moeller Lachnospiraceae	367	250	117	1,337,115	1,170,397	898,401	271,996	23.24%
Nichols Lachnospiraceae	456	326	130	1,331,007	693,009	620,713	72,296	10.43%

^*a*^ “Total reads” is defined as total reads after sequencing. “Total QC reads” is defined as the total reads passing initial QC in the dada2 analysis pipeline. “On-target reads” is defined as reads/ASVs classifying to the bacterial family targeted by the primer. “Off-target reads” is defined as reads/ASVs classifying to any other bacterial family. A breakdown of classified ASVs and total reads are reported summed across all samples tested. Additionally, the “off-target rate” is the percent of off-target reads compared to the total QC reads for each primer set. The Nichols long-read primers consistently show lower off-target amplification rates than the Moeller short-read primers.

**Fig 5:**
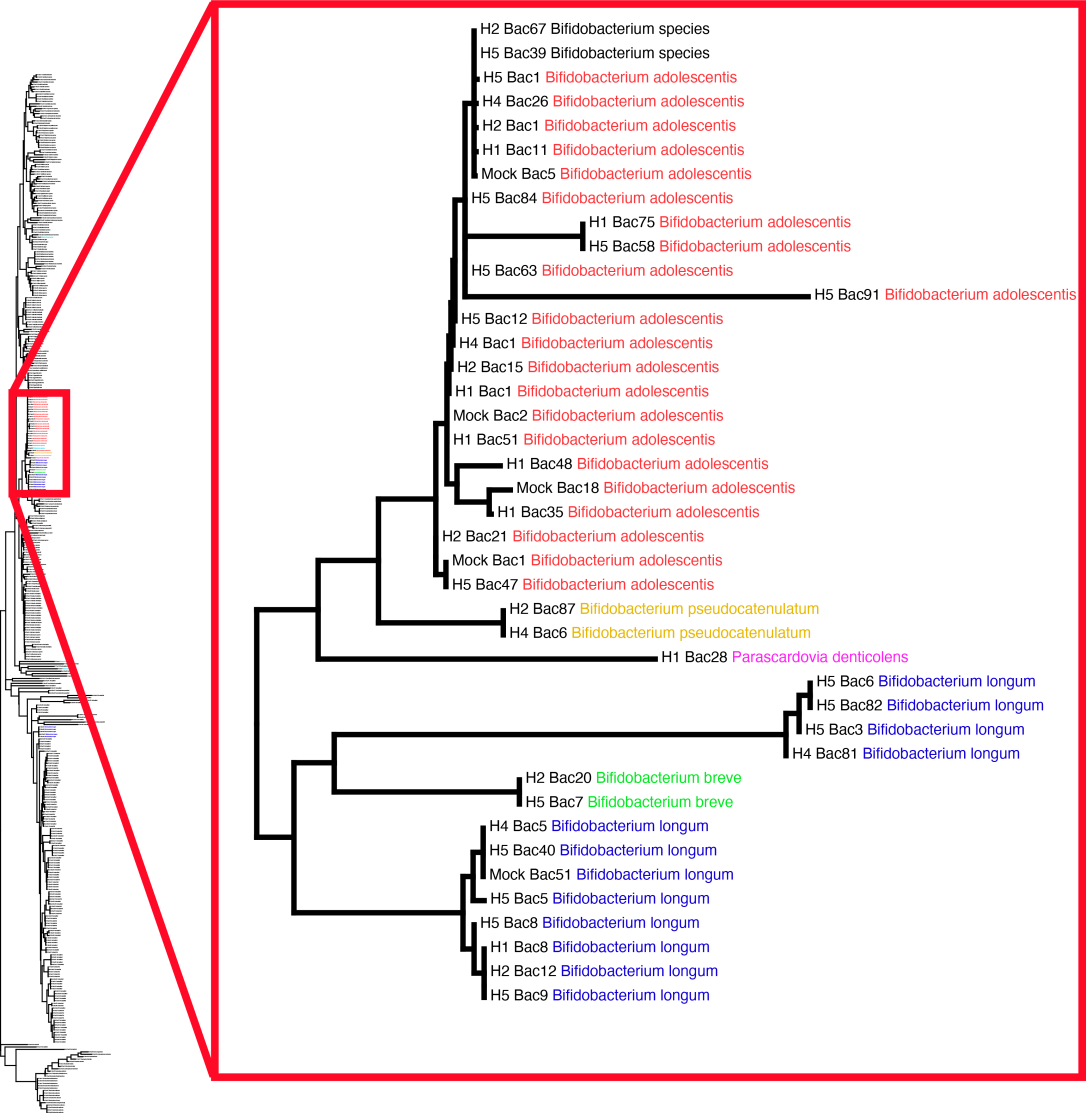
The *gyrB* maximum likelihood tree for Bifidobacteriaceae created from short-read MiSeq ASVs demonstrates extensive off-target amplicons. A total of 256 *gyrB* ASVs were classified from the Bifidobacteriaceae MiSeq reads across the eight samples sequenced. Tips of the tree are ASVs from each sample, labeled first with the sample identifier they came from, the ASV label, and the taxonomic classification of that ASV. ASVs from the same Bifidobacterium species are colored identically for visualization, with off-target ASVs colored in black. The complete maximum likelihood (ML) tree containing all 256 ASVs can be seen on the left. Of the 256 *gyrB* ASVs, only 28 ASVs classified to Bifidobacteriaceae. The majority of the ASVs that classified to Bifidobacteriaceae can be seen in the punch-out on the right.

Using only the forward Moeller Bifidobacteriaceae reads for classification (as was done in Moeller et al. [11]) resulted in 256 ASVs across the five samples that showed amplification (ZymoBIOMICS Gut Microbiome Standard [GM], Human Fecal Sample 1 [H1], Human Fecal Sample 2 [H2], Human Fecal Sample 4 [H4], and Human Fecal Sample 5 [H5]). Of those 256 ASVs, only 28 ASVs (representing 86.32% of total reads) were classified to Bifidobacteriaceae (Fig. 5; Table 2; Table S6). This resulted in a total off-target rate of 13.68%. The Nichols long-read Bifidobacteriaceae primers resulted in 66 total ASVs across the same samples, with 26 ASVs (representing 99.11% of total reads) classifying to Bifidobacteriaceae and a total off-target rate of 0.89% (Table 2; Fig. S5; Table S6). Therefore, the proportion of usable, on-target data were much higher with the Nichols long-read primers (99.11%) vs the Moeller short-read primers (86.32%) for Bifidobacteriaceae.

The Moeller short-read primers for Lachnospiraceae resulted in 367 ASVs across the eight samples. Of the 367 ASVs, only 250 ASVs (76.76% of total reads) were classified to Lachnospiraceae (Table 2; Fig. S6; Table S7). This resulted in a total off-target rate of 23.24%. The Nichols long-read Lachnospiraceae primers resulted in 456 ASVs with 326 ASVs (89.57%) classified to Lachnospiraceae and a total of target rate of 10.43% (Table 2; Fig. S7; Table S7). Therefore, the proportion of usable, on-target data was much higher with the new long-read primers (89.57%) vs the established short-read primers (76.76%) for Lachnospiraceae.

Overall, the Nichols long-read primers result in a lower off-target amplification rate when compared to the Moeller short-read primers. The Nichols Bifidobacteriaceae long-read primers in particular resulted in 99.1% classified ASVs, compared to the 86.3% classification rate with the amplicons created from the Moeller short-read primers, for an increase of 12.8% accuracy.

### 16S data confirm presence of each bacterial family in each sample

16S data were used to confirm the presence or absence of Bacteroidaceae, Bifidobacteriaceae, and Lachnospiraceae in the samples used. We detect ASVs for Bacteroidaceae and Lachnospiraceae in all samples (Table S8). We only see Bifidobacteriaceae ASVs in samples ZymoBIOMICS Gut Microbiome Standard (GM), Human Fecal Sample 1 (H1), Human Fecal Sample 2 (H2), Human Fecal Sample 4 (H4), and Human Fecal Sample 5 (H5) (Table S8). As mentioned above, these five samples were the only ones to show Bifidobacteriaceae *gyrB* amplification as well (Table S6).

Additionally, the ZymoBIOMICS Gut Microbiome Standard was used to evaluate off-target amplifications (Table S9). The Moeller short-read *gyrB* primer sets for Bifidobacteriaceae and Lachnospiraceae generated ASVs not present in the 16S results for the ZymoBIOMICS Gut Microbiome Standard (Table S9). The Nichols long-read Bacteroidaceae *gyrB* primer set had zero off-target amplifications. Both the Nichols long-read *gyrB* primer sets for Bifidobacteriaceae (2 off targets) and Lachnospiraceae (3 off targets) had several off-target amplifications confirmed to be present the 16S results for the ZymoBIOMICS Gut Microbiome Standard (Table S9). None of the Nichols long-read *gyrB* primer sets had additional off-target amplifications (Table S9).

## DISCUSSION

Short-read *gyrB* primers were successfully used in the past to investigate cospeciation between the bacterial members of the gut microbiome and human hosts (11). In the meantime, there have been technological improvements in sequencing that can be used to further elucidate these relationships. Here, we generated and validated primers designed for long-read *gyrB* sequencing targeting the Bacteroidaceae, Bifidobacteriaceae, and Lachnospiraceae families via PacBio sequencing and compared them to the existing short-read *gyrB* primer sets.

Long-read sequencing with PacBio utilizes CCS, which involves sequencing the amplicon multiple times and then taking a consensus to filter out any errors. The PacBio error rate is dependent on how many times the amplicon is sequenced. Since genes like 16S and *gyrB* are relatively small (~1,550 and ~2,500 bp respectively), PacBio can sequence these amplicons 8–10 times via CCS, which equates to an error rate of 0.001% (29). This is superior to the typical error rates in short-read sequencers, which range from ~0.1 to 0.5 (30). So not only does PacBio sequencing provide more genetic information of the gene of interest through increased read lengths, but it also provides a lower error rate than short-read technology. This is beneficial, as single-nucleotide resolution is necessary for evolutionary and coevolutionary studies.

Using long-read sequencing technology by design provides more genetic information because the entire gene of interest can be sequenced rather than just a part of it. The longer reads also provide a more accurate analysis of the gene of interest. The current study illustrated this by comparing the Nichols long-read primers with the Moeller short-read primer sets. Both *gyrB* primer sets were used to sequence the same eight samples (five human samples [H1–H5], two murine samples [M1 and M2], and a mock community sample of known composition [GM]). It should be noted that both the Nichols long-read and the Moeller short-read primers resulted in some off-target amplification, but the rate of off-target amplification was lower for the Nichols long-read primer sets (0%, 0.89%, and 10.43% compared to 1.09%, 13.68%, and 23.24% for the Bacteroidaceae, Bifidobacteriaceae, and Lachnospiraceae families, respectively). Additionally, the Nichols long-read primers showed a higher specificity of the ASVs when compared to the pseudo-MiSeq sequences. On a sample-by-sample basis, the Nichols long-read primers produced a higher percentage of on-target reads when compared to the Moeller short-read primer sets (with the exception of the Lachnospiraceae in the mock sample, which did perform better with the Moeller short-read primer sets).

Long-read *gyrB* sequencing can be a vital tool to complement existing metagenomic methods. A common issue with using metagenomic techniques to assign bacterial taxonomy is an increase of false positive assignments (31). This can prove to be detrimental in downstream analyses of codiversification. Additionally, metagenomic-based codiversification analyses are often limited to only the most common and abundant taxa. For example, in Suzuki et al. (13), only 59 species met inclusion criteria out of the thousands present, leaving major gaps in our understanding of codiversification across more rare or lowly prevalent taxa. We propose that coupling metagenomics with our targeted, clade-specific long-read method would provide a more accurate and reliable view of the species within each family of interest. For example, one could begin by using metagenomic techniques to first identify families of interest that codiversify and then follow up by doing a deeper, targeted analysis of these families with *gyrB* family-specific PacBio primers.

Overall, our newly created long-read *gyrB* primer sets and PacBio library preparation protocol provide a more robust sequencing approach for this non-standard phylogenetic marker gene compared to published short-read sequencing. This improved accuracy and confidence in the classification will enable greater resolution and specificity for cospeciation studies moving forward.

## Data Availability

All sequence data are available on SRA under Bioproject ID PRJNA1055511. All code is available on GitHub at https://github.com/davenport-lab/Long-read_gyrB_method. Reference databases used for the analyses are available on Zenodo under DOIs: 10.5281/zenodo.10451935, 10.5281/zenodo.10452184, and 10.5281/zenodo.10452279. A detailed protocol for generating PacBio libraries is available at protocols.io at dx.doi.org/10.17504/protocols.io.36wgq31nylk5/v1.

## SUPPLEMENTAL MATERIAL

Supplemental Figures msystems.01480-24-s0001.pdf Figures S1-S7.

Supplemental Tables msystems.01480-24-s0002.xlsx Tables S1-S9.

ASM does not own the copyrights to Supplemental Material that may be linked to, or accessed through, an article. The authors have granted ASM a non-exclusive, world-wide license to publish the Supplemental Material files. Please contact the corresponding author directly for reuse.
