# Supplementary material for: Clade-specific long-read sequencing increases the accuracy and specificity of the gyrB phylogenetic marker gene
Source: mSystems. 2024 Dec 16;10(1):e01480-24. doi: 10.1128/msystems.01480-24 (PMC11748558; doi:10.1128/msystems.01480-24)

Supplemental Figure Legends

Supplemental Figure 1: Tanglegram of the *gyrB* maximum likelihood trees created from the Bifidobacteriaceae PacBio ASVs and the Bifidobacteriaceae pseudo-MiSeq ASVs. Bifidobacteriaceae phylogeny was generated using the maximum likelihood (ML) method on ASV data collected across all samples. ML trees for long-read PacBio ASVs (left) and short-read pseudo-MiSeq ASVs (right) were compared with a tanglegram. Each tip is annotated with the sample it originated from, the ASV number, and the taxonomic information after classification. ASV classification names are colored by species. The black lines connecting the two trees illustrate a 1-to-1 relationship where the pseudo-MiSeq ASV results in the same classification as the PacBio parent read. The red dashed lines illustrate a scenario where the pseudo-MiSeq sequence has a less specific classification than the parent PacBio sequence. The blue dashed line represents conflicting classifications between the pseudo-MiSeq ASV and its parent PacBio ASV. The normalized Robinson-Fould distance for these two Bifidobacteriaceae trees was 0.33.

Supplemental Figure 2: Tanglegram of the *gyrB* maximum likelihood trees created from the Lachnospiraceae PacBio ASVs and the Lachnospiraceae pseudo-MiSeq ASVs. Lachnospiraceae phylogeny was generated using the maximum likelihood (ML) method on ASV data collected across all samples. ML trees for long-read PacBio ASVs (left) and short-read pseudo-MiSeq ASVs (right) were compared with a tanglegram. Each tip is annotated with the sample it originated from, the ASV number, and the taxonomic information after classification. ASV classification names are colored by species. The black lines connecting the two trees illustrate a 1-to-1 relationship where the pseudo-MiSeq ASV results in the same classification as the PacBio parent read. The red dashed lines illustrate a scenario where the pseudo-MiSeq sequence has a less specific classification than the parent PacBio sequence. The blue dashed line represents conflicting classifications between the pseudo-MiSeq ASV and its parent PacBio ASV. The normalized Robinson-Fould distance for these two Lachnospiraceae trees was 0.40.

Supplemental Figure 3: The *gyrB* maximum likelihood tree for Bacteroidaceae created from short-read MiSeq ASVs. 82 *gyrB* ASVs were classified from the Bacteroidaceae MiSeq reads. Tips of the tree are ASVs from each sample, labeled first with the sample identifier they came from, the ASV label, and the taxonomic classification of that ASV. ASVs from the same species are colored identically for visualization with off-target ASVs colored in black. Of the 82 *gyrB* ASVs, 55 ASVs classified to Bacteroidaceae.

Supplemental Figure 4: The *gyrB* maximum likelihood tree for Bacteroidaceae created from long-read PacBio ASVs. 31 *gyrB* ASVs were classified from the Bacteroidaceae PacBio reads. Tips of the tree are ASVs from each sample, labeled first with the sample identifier they came from, the ASV label, and the taxonomic classification of that ASV. ASVs from the same species are colored identically for visualization. All 31 *gyrB* ASVs classified to Bacteroidaceae.

Supplemental Figure 5: The *gyrB* maximum likelihood tree for Bifidobacteriaceae created from long-read PacBio ASVs. 66 *gyrB* ASVs were classified from the Bifidobacteriaceae PacBio reads. Tips of the tree are ASVs from each sample, labeled first with the sample identifier they came from, the ASV label, and the taxonomic classification of that ASV. ASVs from the same species are colored identically for visualization. The complete ML tree containing all 66 ASVs can be seen on the left. Of the 66 *gyrB* ASVs, only 26 ASVs classified to Bifidobacteriaceae. Off target ASVs will remain black

Supplemental Figure 6: The *gyrB* maximum likelihood tree for Lachnospiraceae created from the short-read MiSeq ASVs. 367 *gyrB* ASVs were classified from the Lachnospiraceae MiSeq reads. Tips of the tree are ASVs from each sample, labeled first with the sample identifier they came from, the ASV label, and the taxonomic classification of that ASV. ASVs from the same species are colored identically

for visualization, with off-target ASVs colored in black. Of the 367 *gyrB* ASVs, 250 ASVs classified to Lachnospiraceae.

Supplemental Figure 7: The *gyrB* maximum likelihood tree for Lachnospiraceae created from long-read PacBio ASVs. 456 *gyrB* ASVs were classified from the Lachnospiraceae PacBio reads. Tips of the tree are ASVs from each sample, labeled first with the sample identifier they came from, the ASV label, and the taxonomic classification of that ASV. ASVs from the same species are colored identically for visualization, with off-target ASVs colored in black. Of the 456 *gyrB* ASVs, 326 ASVs classified to Lachnospiraceae.

Supplemental Figure 1

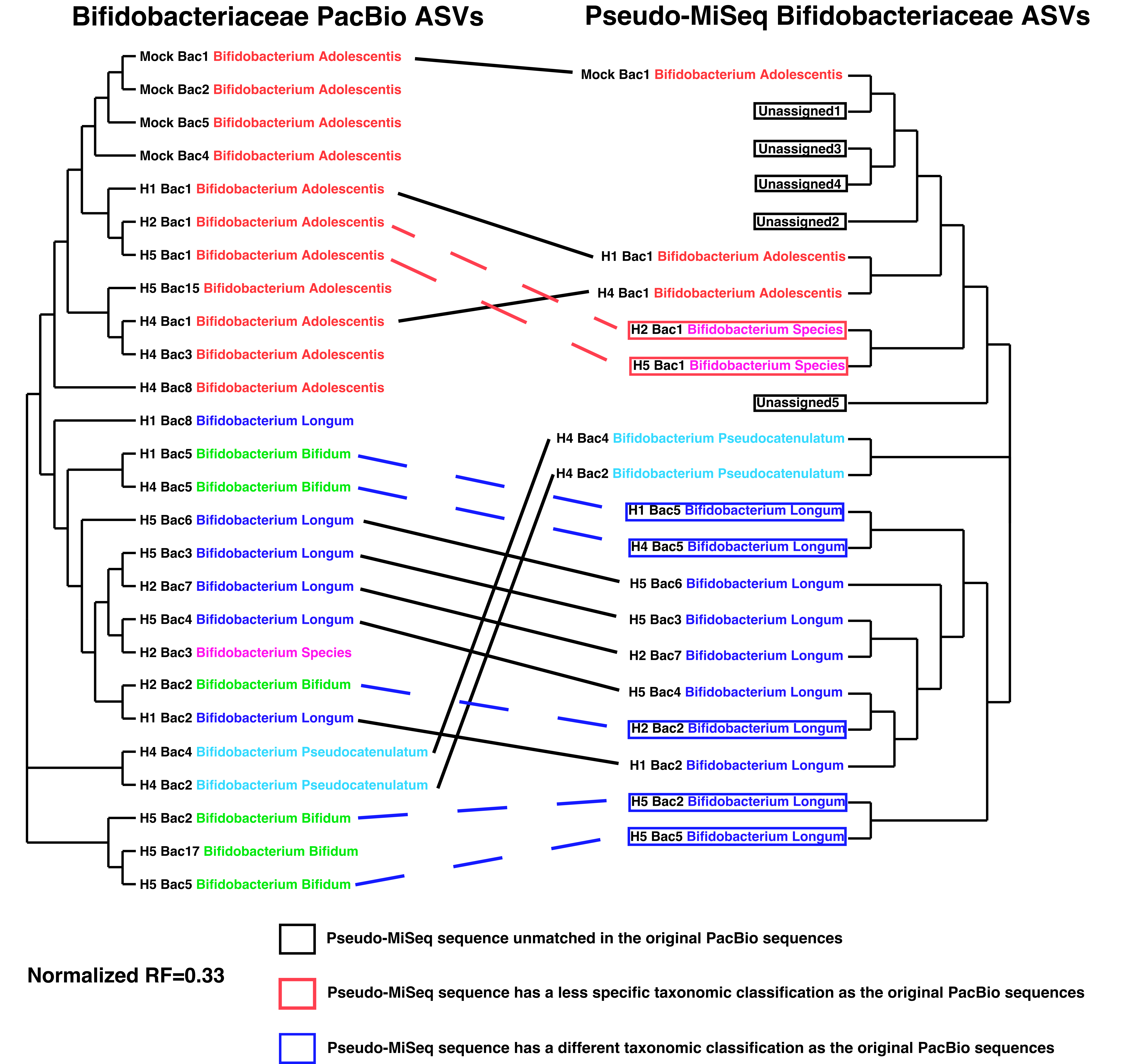

Supplemental Figure 2

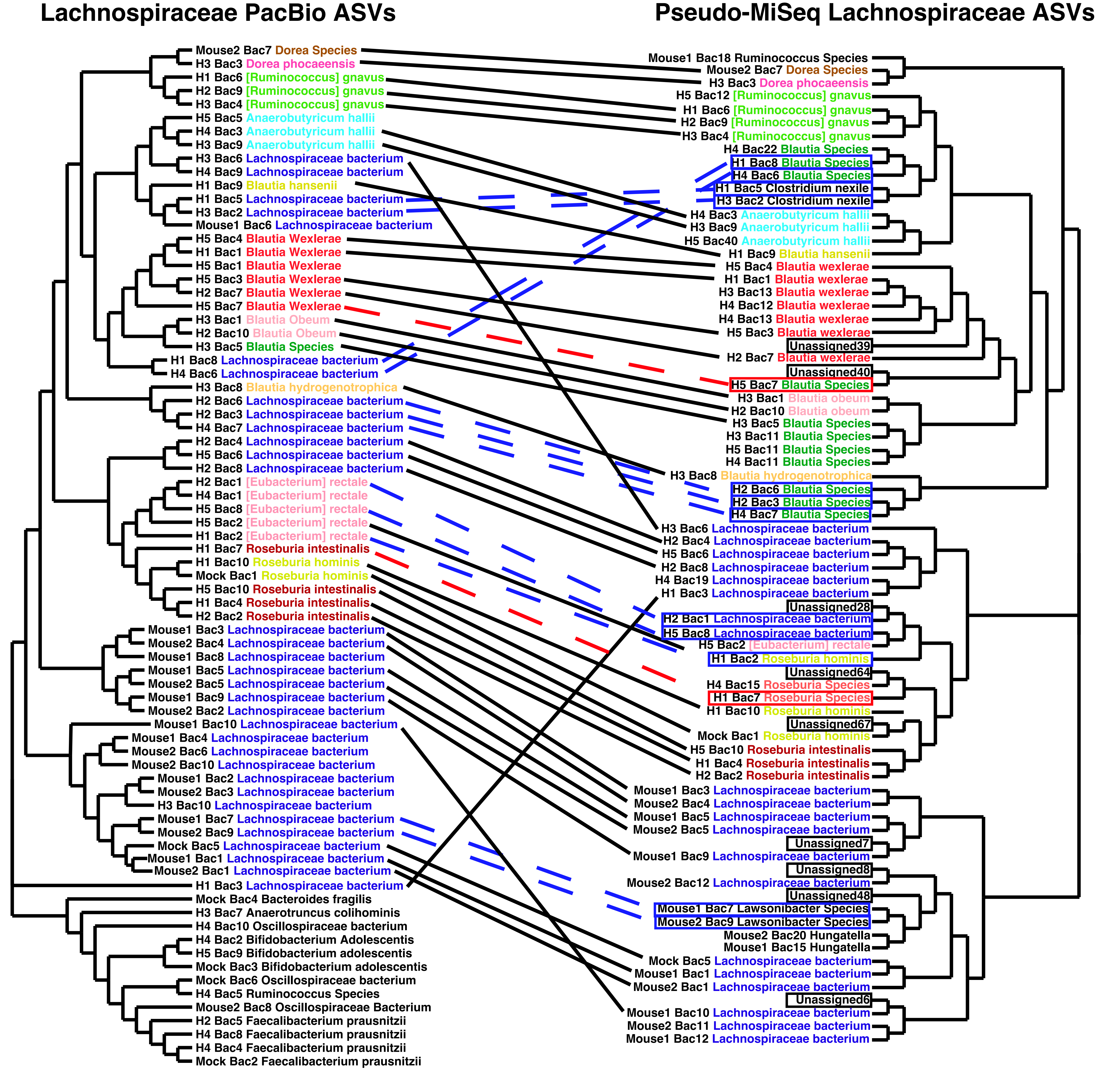

Pseudo-MiSeq sequence unmatched in the original PacBio sequences

Normalized RF=0.40

Pseudo-MiSeq sequence has a less specific taxonomic classification as the original PacBio sequences

Pseudo-MiSeq sequence has a different taxonomic classification as the original PacBio sequences

Supplemental Figure 3

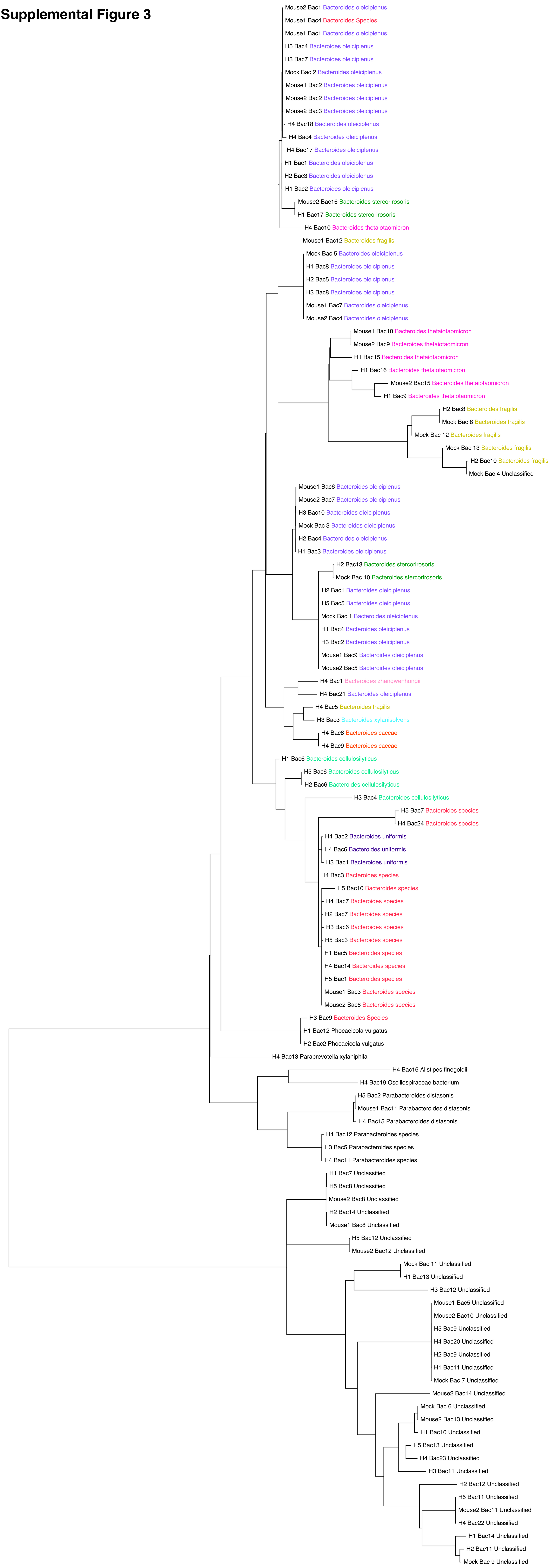

Supplemental Figure 4

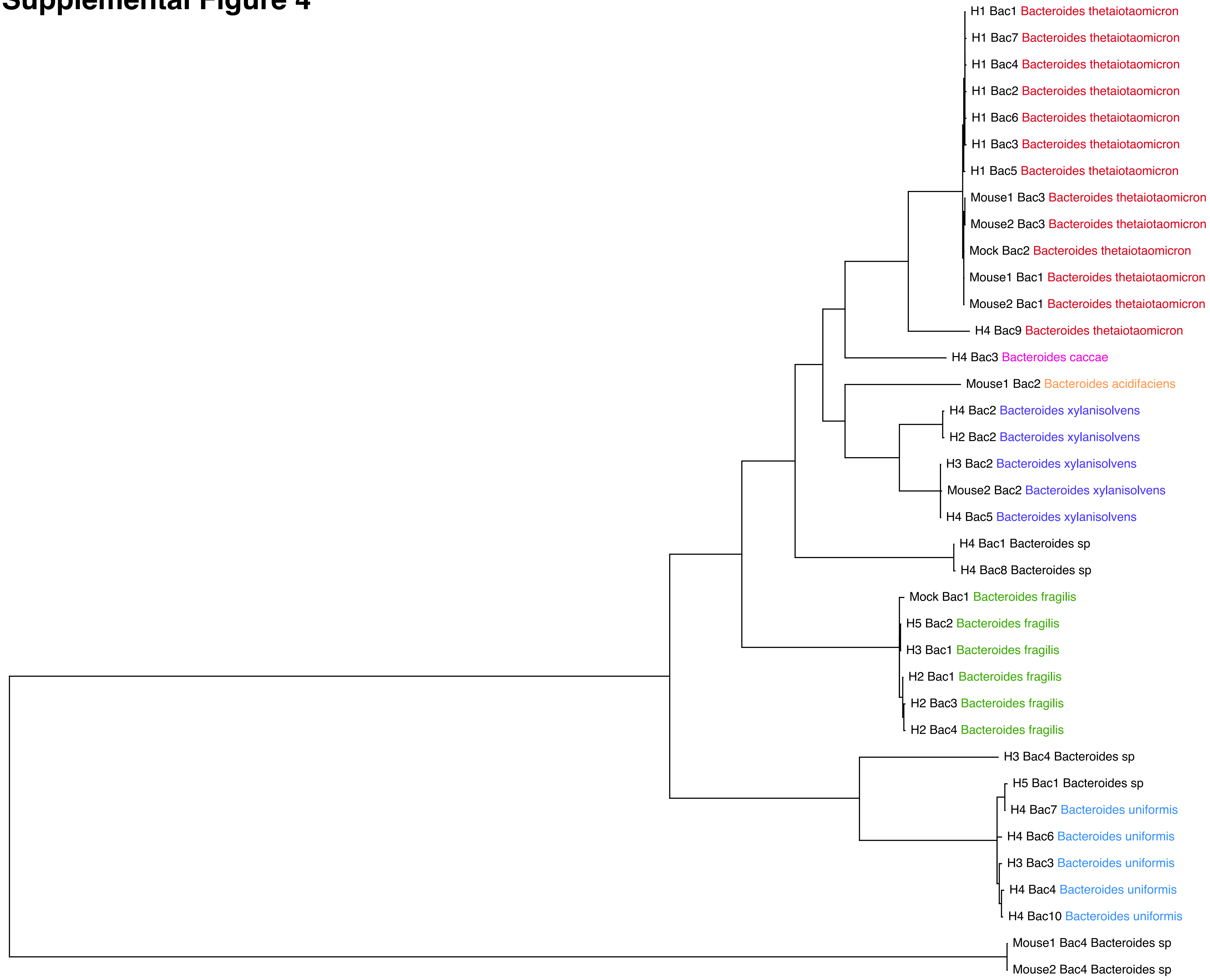

Supplemental Figure 5

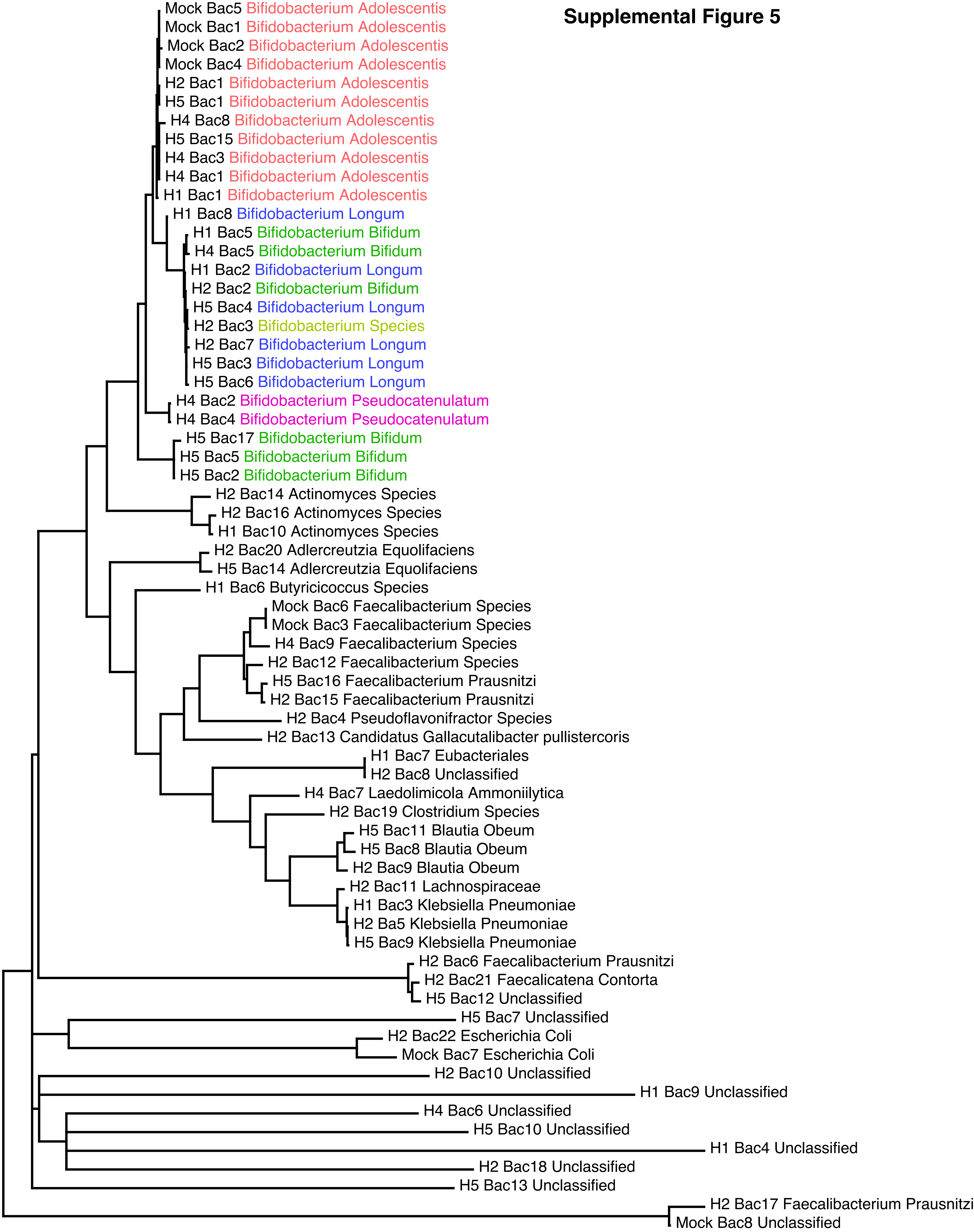

Supplemental Figure 6

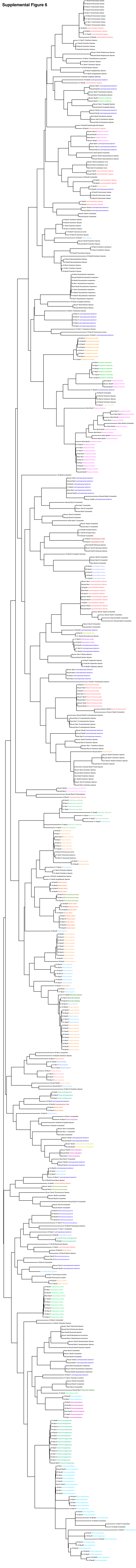

Supplemental Figure 7

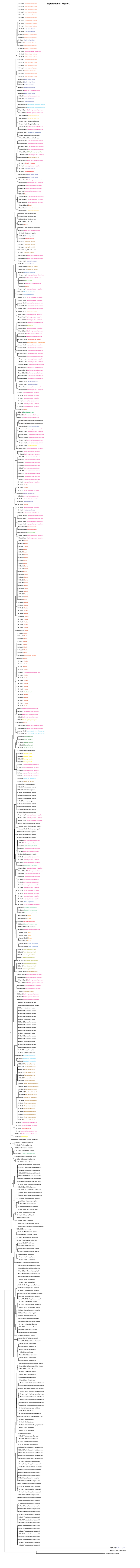

Supplement: Supplemental Figures — Figures S1-S7. [file msystems.01480-24-s0001.pdf]
